# Amine vapor-responsive ratiometric sensing tag based on HPTS/TPB-PVA fluorescent film for visual determination of fish freshness

**DOI:** 10.1016/j.fochx.2024.101152

**Published:** 2024-01-20

**Authors:** Qian Han, Min Yang, Zexin Zhang, Xinwen Bai, Xiuying Liu, Zhenhua Qin, Wei Zhang, Pingping Wang, Lijie Zhu, Zaixi Shu, Xuepeng Li

**Affiliations:** a School of Food Science and Engineering, Wuhan Polytechnic University, Wuhan, Hubei 430028, China; b School of Chemical and Environmental Engineering, Wuhan Polytechnic University, Wuhan, Hubei 430028, China; c Key Laboratory for Deep Processing of Major Grain and Oil, Ministry of Education Wuhan, Hubei 430028, China; d College of Food Science and Technology, Bohai University, Jinzhou, Liaoning 121013, China

**Keywords:** Fluorescent film, Amine vapor, Sensing tag, Fish freshness, Ratiometric indicator

## Abstract

In this study, amine vapor-sensitive films with ratiometric fluorescence attributes were developed. The pH-sensitive fluorescein 8-hydroxypyrene-1,3,6-trisulfonic acid trisodium salt (HPTS) and its tetraphenylethylene derivative (TPB) were selected as ratiometric indicators and incorporated into a polyvinyl alcohol (PVA) matrix to produce HPTS/TPB-PVA films. The films responded well to amine vapors, and the interference of aromatic vapors did not substantially affect the fluorescence signals of the films. Under UV light at a wavelength of 365 nm, the fluorescence of the films changed from dark pink to light pink and finally to yellow when the freshness of the fish was visually checked during storage. In addition, the color difference values of the films showed a positive correlation with the total volatile basic nitrogen (TVB-N), ranging from 12.7 to 24.8 mg/100 g at 25 °C and 8.4 to 25.6 mg/100 g at 4 °C, respectively. This indicates that fluorescent films have good potential for quantifying fish freshness in the near future when connected to an automatic data processing system based on color differences.

## Highlights

• Fluorescent sensing tag was developed for visual detection of fish freshness.
• Sensing tag was based on the HPTS/TPB-PVA film with ratiometric attribute.
• Sensing tag showed dramatic fluorescence color changes during sensing process.
• Sensing tag showed potential for quantitatively predicting of the TVB-N level.

## Graphical abstract

**Figure:**
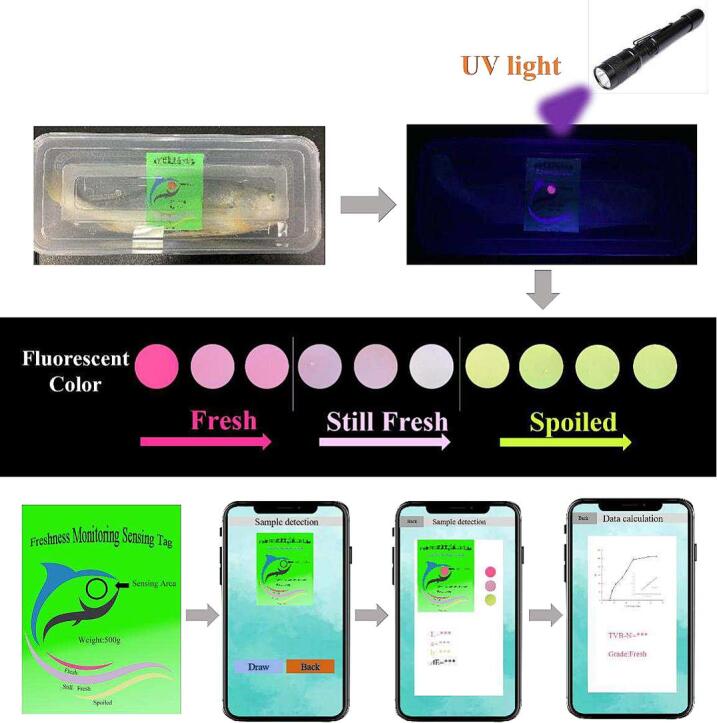

## 1 Introduction

Food spoilage results in food waste. Approximately one-third of the global food supply, equivalent to 1.3 billion tons, is wasted annually (Ma et al., 2021). Monitoring food quality in real-time is essential to satisfy consumer expectations, reduce food waste, and ensure food safety. In particular, aquatic products rich in proteins can easily degenerate during transport and storage, producing harmful substances and negatively impacting consumer health. (Duan et al., 2023, Bondoc and Șindilar, 2002, Bondoc, 2014). However, the evaluation of food freshness relies mainly on the use of instruments. It is difficult for retailers and consumers to identify whether food in packaging is fresh. Therefore, developing a nondestructive, fast, and on-site evaluation method will assist in addressing food waste issues.

Packaging is crucial in preventing food contamination and determining food grade (Hazarika, Konwar, Borah, Saikia, Barman, & Hazarika, 2023). Recently, on-package sensing labels or films, a type of intelligent packaging, has attracted increasing attention. These labels or films offer information on food freshness and edible quality during the entire storage process, showing color or visual signal changes in real time and providing early warnings to consumers (You, Zhang, Wang, Jin, Wei, & Wang, 2022). Among various indicators for developing sensing labels or films, the use of pH indicators in food packaging has been increasing (Amaregouda, Kamanna, & Gasti, 2022). In our previous study, we developed a series of colorimetric labels utilizing pH-sensitive materials, such as bromocresol green pH indicator and polyaniline (Liu et al., 2020, Liu et al., 2020). In packaging, sensing labels that use pH-sensitive dyes typically change color upon interaction with volatile amines (Ezati, Bang, & Rhim, 2021). Consumers assess food freshness based on the color of the sensing label. Nevertheless, there is a need for further improvement in the sensitivity and stability of these colorimetric labels.

The development of intelligent packaging shows considerable potential with the use of fluorescent tags or films that exhibit exceptional sensing capabilities, such as high sensitivity, rapid response, and reversibility (Lai et al., 2021). Generally, fluorescence sensing films are developed based on one type of luminogen, showing variations in fluorescence intensity. However, in certain instances, only a slight change in fluorescence intensity occurs, which is not expected for visual detection (Jia et al., 2019). The sensitivity of single fluorescent signal films used for detection with the naked eye requires further improvement. Ratiometric fluorescent indicators, which integrate the signal strengths of two signal units, can exhibit distinct fluorescent color changes (Chen et al., 2022, Cao et al., 2022, Bigdeli et al., 2019, Gui et al., 2019). These indicators have shown considerable potential for building sensing films because the difference in fluorescent color is most noticeable to the naked eye. Moreover, the ratiometric fluorescence technique can reduce environmental interference and help obtain more accurate detection results (Liu et al., 2021, Yao et al., 2013, Park et al., 2020).

Therefore, in this study, we developed a proportional fluorescence sensing label by combining a pH-sensitive indicator (HPTS) with a reference indicator (TPB), using polyvinyl alcohol (PVA) as the matrix. HPTS, which acts as a sensing indicator, was selected because of its favorable amine-responsive behavior. TPB, a type of aggregation-induced emission-active fluorogen (AIEgen), was selected for its outstanding AIE attributes and high red fluorescence stability, serving as an internal control indicator. The structural characteristics and morphology of the indicator labels were evaluated using Fourier-transform infrared (FT-IR) spectroscopy, X-ray diffraction (XRD), and scanning electron microscopy (SEM). Additionally, we analyzed the physical properties of the indicator labels, including the water vapor transmittance, thickness, mechanical tensile strength, and solubility. Finally, the feasibility of using sensing labels to monitor the freshness of real samples was evaluated.

## 2 Materials and methods

### 2.1 Materials

Polyvinyl alcohol (PVA), HPTS fluorescein, and trimethylamine were purchased from Shanghai Aladdin Biochemical Technology Co., Ltd. (Shanghai, China). Ammonia was obtained from Tianjin Fuchen Chemical Co. (Tianjin, China). TPB were purchased from Xuzhou Dayang Biochemical Technology Co., Ltd (Xuzhou, China). Octanal, ethyl acetate, cyclohexanone and 2-methyl-furan were obtained from Maclin Biochemical Technology Co. Ltd. (Shanghai, China).

Packaged fresh large yellow croakers were purchased from a local market and transported to the lab within a period of 30 min. Before testing, the innards, head, tail, and fins of the fish were removed.

### 2.2 Preparation of HPTS/TPB sensing films

The HPTS/TPB sensing film was produced using a solution casting technique. To begin, 40 mL of deionized water was heated to 100 °C. Then, 2.0 g of PVA was dissolved in the heated water. After cooling to 25 °C, a solution of glutaraldehyde solution (1 mL, 50 wt%) and HPTS (5.0 mg) was added with magnetic stirring for 12 h. Afterward, the above solution was added with TPB-tetrahydrofuran solution (4 mL, 1 mg/mL) to create the film casting solution. To eliminate any bubbles, a petri dish with a diameter of 90 mm was coated with 40 mL of the film casting solution and allowed to dry at 37 °C for 10 h.

### 2.3 Characterization of HPTS/TPB sensing films

#### 2.3.1 Film thickness

The films thickness (mm) was measured using a hand-held digital micrometer (SL01-22, Biaokang, China), at 5 random locations on each piece of the film.

#### 2.3.2 Scanning electron microscope (SEM)

The surface and internal structure of the film samples were characterized using a SEM (SU8020, Hitachi, Japan**)**. The observations of the samples were performed under a 3.0 kV accelerating voltage.

#### 2.3.3 Fourier transform infrared (FTIR) spectroscopy

FTIR spectra of the films were obtained using a FTIR spectrometer (Scimitar 2000, Varian, USA). The film samples were scanned in the range of 400 ∼ 4000 cm^−1^ with a resolution of 4 cm^−1^. Each sample was scanned 32 times.

#### 2.3.4 X-ray diffractometer (XRD)

The changes in crystalline properties of the HPTS/TPB sensing films were assessed using a XRD diffractometer (UItima IV, Rigaku, Japan). The readings were conducted with 2*θ* ranging from 5° to 55°, operating at 40 kV and 40 mA. The detection rate was set at 0.05°/s.

### 2.4 Physical properties of HPTS/TPB sensing films

#### 2.4.1 Water solubility (WS)

The WS was tested using the method as reported (Li, Liu, Ye, Wang, & Wang, 2015). The solubility of the sensing films was calculated using the following equation:

$$WS\left(\%\right)=\frac{m_{1}-m_{0}}{m}*100\%$$

where “m_0_” represents the starting dry mass, and “m_1_” is the ending dry mass.

#### 2.4.2 Water vapor permeability (WVP)

The WVP was assessed following the American standard test methods (ASTM E96M-05, 1995) with modification. Briefly, a testing beaker was filled with 3 g of calcium chloride and sealed using the film sample. The beaker was stored at 25 °C (75 % RH), and then measured after 24 h.

The WVP was calculated using the following equation (Cao et al., 2022, Chen et al., 2022):

$$\mathrm{WVP}=\frac{d\times \Delta M}{S\times \Delta P}$$

where *Δ*M is the mass difference of the beaker (g) after 24 h, d is the film thickness (mm), S is the covered area of the film (m^2^), and *Δ*P (Pa) is the partial pressure of vapor.

#### 2.4.3 Water contact angel (WCA)

To determine the WCA, a droplet of 1 μL ultrapure water was deposited onto the film surface, and measured using a WCA analyzer (OCA25, Dataphysics, Germany) (Yang et al., 2023). The change in WCA was recorded to analyze the surface hydrophobicity.

### 2.5 Functional properties of HPTS/TPB sensing films

The fluorescence response of the HPTS/TPB sensing films to amine vapor, and vapor of aromatic chemicals was determined according to the previous research (Liu et al., 2020, Liu et al., 2022). Briefly, the sensing films were placed in the headspace of the 100 mL 0.01 % trimethylamine, 0.01 % dimethylamine, and 0.01 % ammonia, respectively. Images of the sensing films under 365 nm ultraviolet light (UVSABIP, Puxi, Beijing, China) were recorded using a smartphone every 30 min. For the volatile aromatic vapor test, aromatic chemicals including 2-methyl-furan, ethyl acetate, cyclohexanone, and octanal were selected and tested using the same method.

### 2.6 Fish freshness evaluation during storage

#### 2.6.1 Total viable counts (TVC)

TVC was measured following the Chinese standard GB 4789.2 with some modification. Briefly, 10 g of sample and 90 mL of sterile physiological saline were placed in a sterile bag, and gently patted for 1 ∼ 2 min to prepare a 1:10 diluted sample solution. The sample solution was further diluted to 1:100, and 1 mL of the sample dilution was injected into a sterilized agar plate, mixed with 15 ∼ 20 mL of plate counting agar medium. The plate was then incubated at 30 °C, and the number of colonies was record after 72 ± 3 h. The result was presented as logarithm of colony forming units (CFU) per gram.

#### 2.6.2 Total volatile basic nitrogen (TVB-N)

The determination of TVB-N value followed the method described in GB 5009.228–2016 with slight modifications. In summary, 5 g of the sample was mixed with 50 mL of deionized water and homogenized. The homogenized sample was then filtered, and the filtrate was collected. In the outer chamber of the diffusion dish, 1 mL of the filtrate and 1 mL of saturated potassium carbonate solution were added. In the inner chamber of the diffusion dish, 1 mL of 20 g/L boric acid solution and a mixture of bromocresol green and methyl red indicators were added. After sealing the diffusion dish, it was incubated at 37 °C for 2 h. The inner chamber filtrate was then titrated with 0.01 mol/mL hydrochloric acid until a purple-red color was observed. The volume of hydrochloric acid consumed during the titration was recorded, and the TVB-N content was calculated.

#### 2.6.3 Thiobarbituric acid (TBA)

The detection of TBA followed the method described in GB/T 35252–2017. In summary, 5 g of the sample was mixed with 50 mL of 7.5 % trichloroacetic acid, homogenized, and allowed to stand for 30 min. The mixture was then filtered, and the filtrate was collected. Next, 5 mL of the filtrate was transferred into a test tube, and 5 mL of 0.02 mol/L 2-thiobarbituric acid solution was added. The mixture was placed at 90 °C for 30 min and then measured at 532 nm. The result of the measurement was reported as mg malonaldehyde (MDA) per kilogram.

#### 2.6.4 pH measurement

According to the Chinese standard GB 5009.237, the pH values of fish samples were determined. In general, 5 g of the sample was mixed with 50 mL of a 0.1 mg/mL potassium chloride solution and homogenized. The pH measurements were then carried out using a digital pH meter. Three replicate measurements were performed.

### 2.7 Utilization of the films for visual assessing of fish freshness

Fish spoilage trials were performed at room temperature (25 °C) and chilled temperature (4 °C). Briefly, 200 g of fresh large yellow croaker samples were placed in PP food grade plastic boxes (20 × 10 × 5 cm). Sensing films with a diameter of 1.5 cm were attached to the inside of the top of each box. In order to facilitate interactions between the films and vapors, four sticky spacers measuring 0.5 cm thick were placed at the four corners of the tags, creating a gap with the cap. Throughout the experiment, it was ensured that the plastic sample boxes remained sealed. The fluorescence images of the sensing labels were captured by exposing the sample box to 365 nm ultraviolet light. Under storage conditions of 25 °C, the images were recorded once every 4 h, while under storage conditions of 4 °C, the images were recorded once every 2 days. All experiments were conducted in triplicate.

### 2.8 Data analysis

The R, G, and B values were obtained by reading three spots located on the upper, middle, and lower parts of each sensing label. The color change sensitivity (*S*) was processed according to the following equation (Huang, Zou, Zhao, Shi, Li, & Shen, 2015):

$$S\left(\%\right)=\frac{|R-R_{0}|+|G-G_{0}\left|+\right|B-B_{0}}{R+G+B}\times 100\%$$

where, R_0_ (red), G_0_ (green) and B_0_ (blue) were the starting values of the images, and R, G, and B were the values at the sampling point.

The total color difference (*Δ*E) was determined according to the following equation (Chen, Zhang, Bhandari & Yang, 2020):

$$\Delta E=\sqrt{{\left(L-L_{0}\right)}^{2}+{\left(a-a_{0}\right)}^{2}+{\left(b-b_{0}\right)}^{2}}$$

where, L_0_, a_0_ and b_0_ were the initial lightness, redness, and yellowness values collected, and L, a, and b were the corresponding values at the sampling point.

The color data, including R, G, B, L, a, and b values, were extracted from the captured images using the Adobe Photoshop 2021 software. The data were then processed using Origin 2018 software.

## 3 Results and discussion

### 3.1 Characterization of the HPTS/TPB-PVA sensing film

**Scheme 1:**
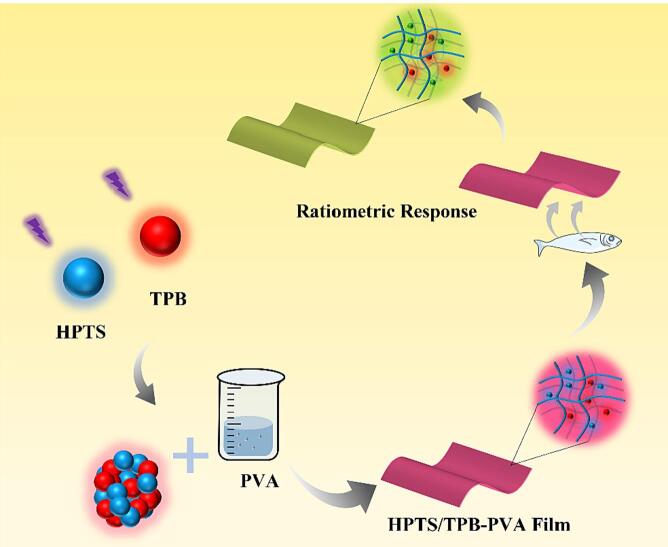
Schematic representation of amine response of the film.

The HPTS/TPB-PVA film was developed based on the HPTS/TPB ratiometric indicator; a schematic representation is shown in Scheme 1. The red fluorescent reference dye TPB and indicator dye HPTS were immobilized in PVA to prepare the indicator films. The sensing film initially exhibited pink fluorescence and then interacted with the volatile amines released from the sample, causing a change in the fluorescence signal of the sensing dye, TPB. This ultimately resulted in a shift in the fluorescence color of the sensing film from pink to yellow.

**Fig. 1:**
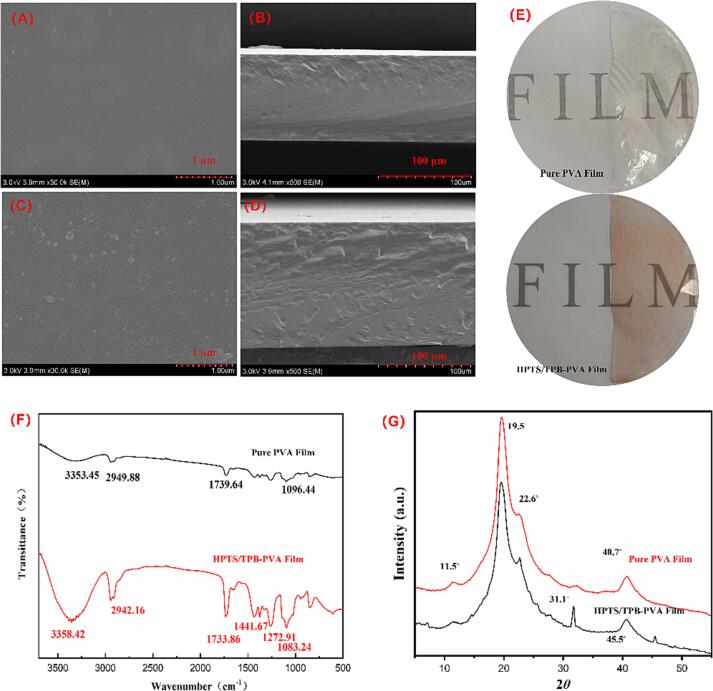
SEM images of surfaces and cross-sections of (A, B) PVA film, and (C, D) HPTS/TPB-PVA film; (E) visual appearance of the films; (F) FTIR curves of pure PVA and HPTS/TPB-PVA film; (G) XRD curves of pure PVA and HPTS/TPB PVA film.

SEM images of the PVA and HPTS/TPB-PVA films are shown in Fig. 1. The PVA film exhibits a smooth, dense, and even surface. After the addition of HPTS/TPB, small raised particles were observed owing to HPTS/TPB aggregates. Thus, it can be concluded that the embedding of HPTS/TPB did not have a notable impact on the film surface. However, the cross-section of the HPTS/TPB PVA films was rougher than that of the PVA film. This phenomenon can be explained by the interaction between the HPTS/TPB and the PVA matrix. Compared to the pure PVA film, the visual aspect of the HPTS/TPB-PVA film presented a slightly pinkish color. (Fig. 1 E). The thickness of different batches of HPTS/TPB sensing films was measured, and results ranged between 0.183 ± 0.018 to 0.247 ± 0.015 mm.

Fig. 1 F shows the FTIR spectra of the PVA and HPTS/TPB PVA films. The peaks at approximately 3353, 2949, 1739, and 1096 cm^−1^ indicate –OH, –CH, H—O—H stretching, and C—O stretching, respectively (Cao et al., 2022, Chen et al., 2022, Zhou et al., 2023). These four peaks were observed in both spectra. After embedding HPTS/TPB, the C—O peak shifted from 1096 to 1083 cm^−1^, representing changes in C—O stretching. The peaks at 1733 (H—O—H stretching) and 1083 cm^−1^ (C—O stretching) of the HPTS/TPB-PVA spectra were sharper, verifying the interaction between HPTS/TPB and PVA. The characteristic absorption peaks at 1441 and 1272 cm^−1^ were attributed to C—O specific angular deformation and C—H wagging, respectively (Liu et al., 2023, Al-Muntaser et al., 2022).

Fig. 1 G showed that the XRD pattern of the HPTS/TPB-PVA film was different from that of the original PVA film. The original PVA film exhibited four characteristic diffraction peaks at 11.5°, 19.5°, 22.6°, and 40.7°, corresponding to indices planes (1 0 0), (1 0 1), (2 0 0), and (1 1 1), respectively. This indicated that the PVA film possessed a semicrystalline structure, which is attributed to the strong intermolecular hydrogen bonding interaction within PVA chains (Al-Muntaser et al., 2022). In the XRD curve of HPTS/TPB-PVA film, two new diffraction peaks at 31.1° and 45.5° appeared. From this, it can be concluded that the uniformity of the film was altered, and the structured layout of PVA was disrupted as a result of the interaction between HPTS, TPB, and PVA.

### 3.2 Physical properties of HPTS/TPB-PVA sensing films

The hydrophilic and hydrophobic properties were characterized based on WCA and WS measurements. The WCA of the PVA films is 21.8°. After embedding HPTS/TPB, the WCA of the HPTS/TPB-PVA sensing film changed to 64.5°. This indicated that the hydrophilic properties of HPTS/TPB-PVA were inferior to those of pure PVA. This phenomenon may be attributed to the incorporation of TPB molecules, which changed the cross-linked structure of pure PVA, ultimately restricting the movement of –OH on the PVA film (Feng, Xu, Shao, Zhu, Qiu, & Zhu, 2022).

In Table S1, the results presented that the WS of the pure PVA and HPTS/TPB PVA films were 21.37 % and 3.45 %, respectively. Owing to the hydrophobicity of the basic component, TPB, in the film, the WS of the HPTS/TPB PVA film decreased by approximately seven-fold. Notably, the lower WS of the HPTS/TPB PVA composite film could prevent the interference caused by water vapor in the package during the sensing process, which is a good attribute for developing the sensing tag.

The WVP of pure PVA and HPTS/TPB-PVA were measured to evaluate the water molecule diffusion properties of the films. As shown in Table S1, the WVP values of HPTS/TPB-PVA were lower than those of pure PVA. The WVP values decreased by 19.85 % (from 0.403 ± 0.05 to 0.323 ± 0.08 g.mm/m^2^Kpa). This indicates that incorporating the HPTS/TPB indicator could decrease the WVP of PVA.

From the above results, it can be found that compared to packaging materials such as starch and chitosan, PVA has excellent physical properties and is a good choice for the preparation of freshness indicator labels. This is consistent with the findings of Zhang et al. (Zhang, Sun, Sang, Jia & Ou, 2022).

### 3.3 Functional properties of HPTS/TPB-PVA sensing films

**Fig. 2:**
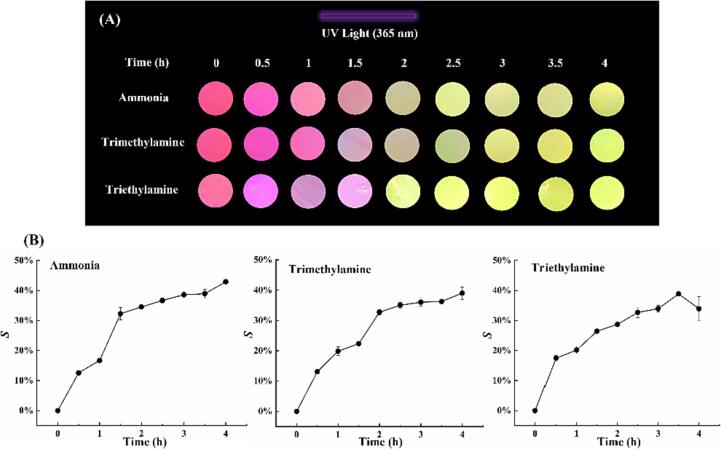
(A) Fluorescence changes, and (B) *S* value trends of the sensing films in ammonia environment.

Amine vapor, including ammonia, trimethylamine, and triethylamine, is produced during fish degradation (Bondoc, 2016, Bondoc, 2016b). To verify the response of the films to amine vapors, we first determined the fluorescence color changes of the films exposed to the headspaces of different amine solutions at the same concentration. As shown in Fig. 2, the fluorescence signal of the HPTS/TPB-PVA films gradually changed from pink to yellow, and the color could be detected with the naked eye. The same trend was observed in the S value analyses. The S values increased sharply to approximately 30 % before 1.5 h and then increased gradually from 30 % to 40 % after 1.5 h. It was inferred that the sensing films exhibited acceptable responses to the different amine vapors generated during fish storage.

The dynamic color-change process of the HPTS/TPB-PVA sensing film was recorded for approximately 4 min; the results are shown in Supplementary Video 1 (20 × speed processing). The sensing film showed fluorescent color changes within approximately 3 min of dropping 0.01 % ammonia solution onto the surface of the film.

**Fig. 3:**
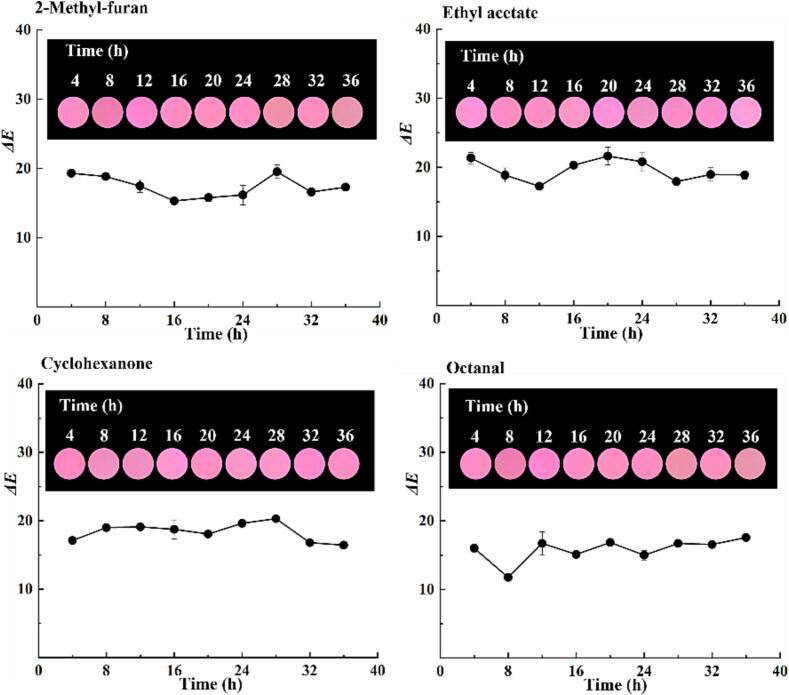
*Δ*E value trends of the sensing films in flavor vapors, and the fluorescent images of the films (insert).

During fish storage, various flavor vapors are generated in the sealed trays and may interfere with film sensitivity (Martin, Joly, Dupas-Farrugia, Adt, Oulahal, & Degraeve, 2023). The responses of the films to several representative flavor vapors were analyzed, and the results are shown in Fig. 3. As the exposure time of the films to flavor vapors increased, no pronounced changes in color were observed. The fluorescent color of the sensing films is pink to the naked eye. The trends in the S values were also analyzed, and no notable changes in the S values were observed during the entire exposure process. This verified that the films were good candidates for monitoring fish freshness owing to their high selectivity.

### 3.4 Freshness indicators assay

Changes in fish freshness based on freshness indicators were tested during storage, and the trends of these indicators are presented in Fig. S1 and Fig. S2. As shown in Fig. S1 A and Fig. S2 A, changes in pH values during the storage (25 °C and 4 °C) showed a similar downward trend at the beginning, followed by an upward trend. The potential reason for the decline in pH could be the dissolution of carbon dioxide within the fish muscle, whereas the increase in pH was likely due to the accumulation of volatile bases (Chaijan, Benjakul, Visessanguan, & Faustman, 2005).

TBA is a frequently employed indicator of lipid oxidation in fish muscle (Li, Li, & Hu, 2013). As demonstrated in Figure S1 D and Fig S2 D, there was a progressive increase in the TBA values with increasing storage time. Because of the absence of clear TBA limit standards in the aquaculture industry, incorporating other freshness indicators is essential to assess the freshness of samples comprehensively.

To evaluate the extent of protein decomposition, TVB-N was used as a freshness indicator (Zhang et al., 2023). In Fig. S1 B, the rate of TVB-N formation increased gradually before 20 h and then increased rapidly from 20 to 36 h at the storage temperature of 25 °C. When considering a level of 25 mg/100 g as the threshold of spoilage (Li, Hu, Li, Zhang, Zhu, & Li, 2012), TVB-N values reached from the starting value of 6.52 mg/100 g to 24.85 mg/100 g at 24 h, indicating the shelf life of the fish sample was 24 h. Correspondingly, in Fig. S2 B, the TVB-N values increased progressively and reached the upper limit on the 10th day at 4 °C.

As shown in Fig. S1 C, TVC was relatively low in the initial 8 h (storage at 25 °C) and 4 d (storage at 4 °C), respectively. An apparent increase was observed during storage. The TVC counts reached 6.56 and 6.38 log CFU/mL at 20 h and on the 8th day, respectively, which were close to the acceptable limit of 7 log CFU/mL for marine species (Özyurt et al., 2009, Raeisi et al., 2017). These results indicated that the spoilage limit of TVC was slightly shorter than that of TVB-N during storage.

Based on the above results of these freshness indicators, the threshold of spoilage of the fish sample in this study is 20 h at 25 °C and 8 d at 4 °C.

### 3.5 Application of the HPTS/TPB-PVA sensing films

The Indicating label was attached to the inner surface of the packaging to monitor the freshness of the samples under two temperature conditions: 25 and 4 °C. These two levels were chosen for testing because fish are typically stored and sold under these conditions.

**Fig. 4:**
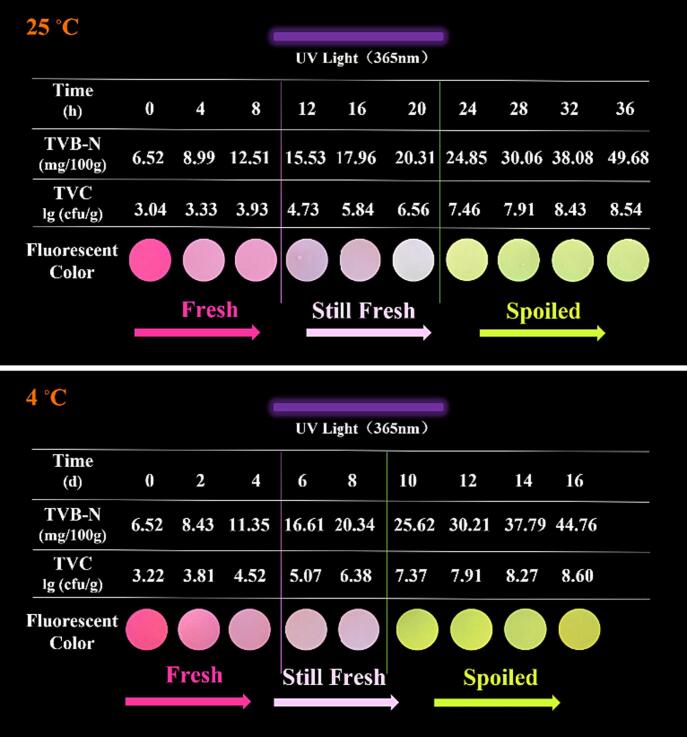
Freshness monitoring of the HPTS/TPB-PVA film at different storage conditions.

The fish sample was initially monitored for freshness at 25 °C. The TVB-N and TVC levels in the fish steadily increased with prolonged storage. Simultaneously, the fluorescence of the HPTS/TPB-PVA film slowly changed from pink to light pink and finally turned yellow. A relationship between the TVB-N content and color changes was observed. As shown in Fig. 4, the TVB-N content was initially low, and the indicator label appeared pink. After a storage time of 12 h, the TVB-N content increased to 15.53 mg/100 g. The pink color of the film gradually faded, indicating that the quality of the fish samples was within acceptable limits. When the storage time was 24 h, the content of TVB-N increased to 24.86 mg/100 g, and the film became completely yellow, indicating that the fish had reached a threshold where it was no longer suitable for consumption. TVC results indicated that The TVC content was less than 7 mg/100 g before 20 h, which is below the TVC standards for edible fish (Zhao, Li, Wang, & Lv, 2012).

Fish spoilage trials were also performed at chilled temperatures (4 °C). In Fig. 4, the TVB-N at the starting point was 6.52 mg/100 g and then slowly escalated to 16.61 mg/100 g on the 6th day. It reached 25.62 mg/100 g on the 10th day, exceeding the limit for spoiled fish (Ruiz-Capillas & Moral, 2001). The fluorescence of the HPTS/TPB-PVA film changed from dark pink to light pink on the 6th day and then to yellow after 8 d, indicating that the fish sample spoiled. These results indicate that the HPTS/TPB-PVA sensing films showed a dramatic color change to the naked eye at the spoilage point. This proves that the HPTS/TPB sensing film is a good candidate for intelligent packaging materials and has the potential for application in the visual monitoring of fish freshness.

### 3.6 Packaging test

**Fig. 5:**
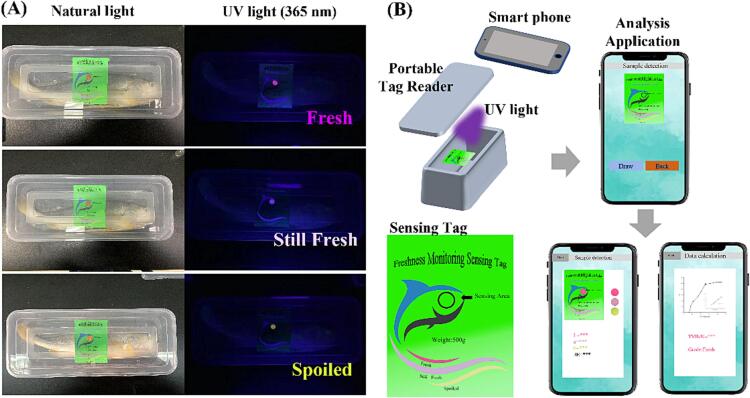
(A) Fluorescence change of HPTS/TPB ratio indicator label in actual samples and (B) the conceptual portable tag reader.

To verify the fluorescence properties and color changes of the HPTS/TPB film in a real package, the films were designed and processed as intelligent sensing tags and attached to commercial packaging of large yellow croaker fish samples. As shown in Fig. 5 A, a clear fluorescence color change from pink to yellow was observed through the caps of the packages when the fish packages were placed under UV light at 365 nm. A conceptual portable tag reader was also designed for intelligent monitoring of fish freshness in real time for retailers and consumers. As shown in Fig. 5 B, the tag reader consists of UV light, a photo window, and a data processing system that can connect to personal smartphones. The phone camera was utilized to collect photos of the sensing tag directly, and detailed data were processed automatically. A portable tag reader is also an ideal unit for integration with intelligent transportation vehicles, providing fresh data instantly during transportation.

### 3.7 Accuracy evaluations

For the intelligent development of the sensing tag technology, the *Δ*E values were processed based on the images and fit with the content of TVB-N. A linear fitting equation can be used to predict the TVB-N levels during the fish spoiling process quantitatively. As shown in Fig. S3, at the storage temperature of 25 °C, the linear fitting equation was y = 4.587x-19.959 within the TVB-N concentrations ranging from 12.7 to 24.8 mg/100 g, with R^2^ = 0.991. Correspondingly, at the storage temperature of 4 °C, the calibration equation was described as y = 4.1712x-12.105, with R^2^ = 0.990, in the range of 8.4 to 25.6 mg/100 g.

To validate the accuracy of the predicted results, the TVB-N content throughout the storage stages was measured using a control method (GB/T 5009.228). The predicted TVB-N content was calculated using the *Δ*E values extracted from the images of the sensing film at various stages. Statistical comparison was performed between the predicted results obtained using the developed sensing film and those obtained using the control method. When the sensing film exhibited a light pink color, the predicted TVB-N values ranged from 14.390 to 15.774 mg/100 g. By contrast, when the sensing film turned yellow, the predicted values of TVB-N ranged from 24.685 to 25.150 mg/100 g for three different batches of real samples (see Table S2). Statistical comparisons suggested no statistically significant differences between these two results, indicating that accurate results could be obtained based on the linear fitting equation of the color change data. The fabricated HPTS/TPB film can potentially be used for the visual measurement of fish freshness and quantitative prediction of TVB-N levels without tedious sample pretreatment.

## 4 Conclusions

In this study, ratiometric fluorescent films were successfully developed for visual monitoring of fish freshness. The pH-sensitive indicator HPTS was selected as an indicator of amines generated from fish spoilage, whereas TPB, with AIE properties and highly stable red fluorescence, was selected as an internal control indicator. The HPTS/TPB ratiometric fluorescent indicator was integrated into the PVA matrix to obtain HPTS/TPB-PVA films. The films exhibited homogeneous surface structures. The incorporation of HPTS/TPB reduced the WS and WVP of the films, which is beneficial for avoiding interference with water vapor in the package. Under the stimulation of amines released from the fish samples, the sensing films exhibited distinct fluorescent color changes from pink to yellow. Furthermore, there is a linear relationship between the concentration of TVB-N and the *Δ*E values of the sensing films under storage conditions of 25 and 4 °C. Therefore, consumers or retailers can assess the freshness of fish not only visually but also accurately determine the TVB-N level by analyzing the *Δ*E values extracted from sensing film images. This provides a convenient method for analyzing fish freshness.

## CRediT authorship contribution statement

**Qian Han:** Investigation, Writing – original draft. **Min Yang:** Investigation, Writing – original draft. **Zexin Zhang:** Writing – original draft, Methodology, Investigation, Conceptualization. **Xinwen Bai:** Formal analysis. **Xiuying Liu:** Writing – original draft, Data curation, Conceptualization. **Zhenhua Qin:** Conceptualization, Writing – review & editing. **Wei Zhang:** Data curation. **Pingping Wang:** Writing – review & editing. **Lijie Zhu:** Writing – review & editing. **Zaixi Shu:** Software. **Xuepeng Li:** Funding acquisition.

## Declaration of competing interest

The authors declare that they have no known competing financial interests or personal relationships that could have appeared to influence the work reported in this paper.

## Data Availability

No data was used for the research described in the article.
