# Supplementary material for: Amine vapor-responsive ratiometric sensing tag based on HPTS/TPB-PVA fluorescent film for visual determination of fish freshness
Source: Food Chem X. 2024 Jan 20;21:101152. doi: 10.1016/j.fochx.2024.101152 (PMC10850885; doi:10.1016/j.fochx.2024.101152)
Supplement: Supplementary data 1 [file mmc1.docx]

Table S1 Physical parameters of (HPTS/TPB) sensing films

| **Physical properties** | **Pure PVA Film** | **HPTS/TPB-PVA Film** |
| --- | --- | --- |
| WCA (°) | 64.5 | 21.8 |
| WS (%) | 21.37±0.04 | 3.45±0.01 |
| MVP (g.mm/m^2^.Kpa) | 0.403±0.05 | 0.323±0.01 |

Table S2 Freshness monitoring of different batches of fish samples based on the sensing tags

| Sample Batch | Control  TVB-N（mg/100g） | Predicted  TVB-N（mg/100g） | Fluorescence Signal of the sensing tags |
| --- | --- | --- | --- |
|  | 6.843±0.536 | - | 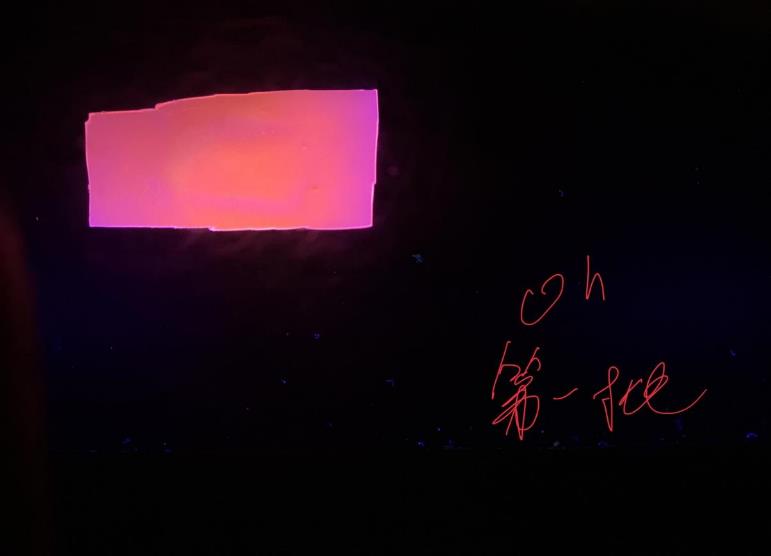 |
| 1 | 14.390±0.219 | 15.284±0.145 | 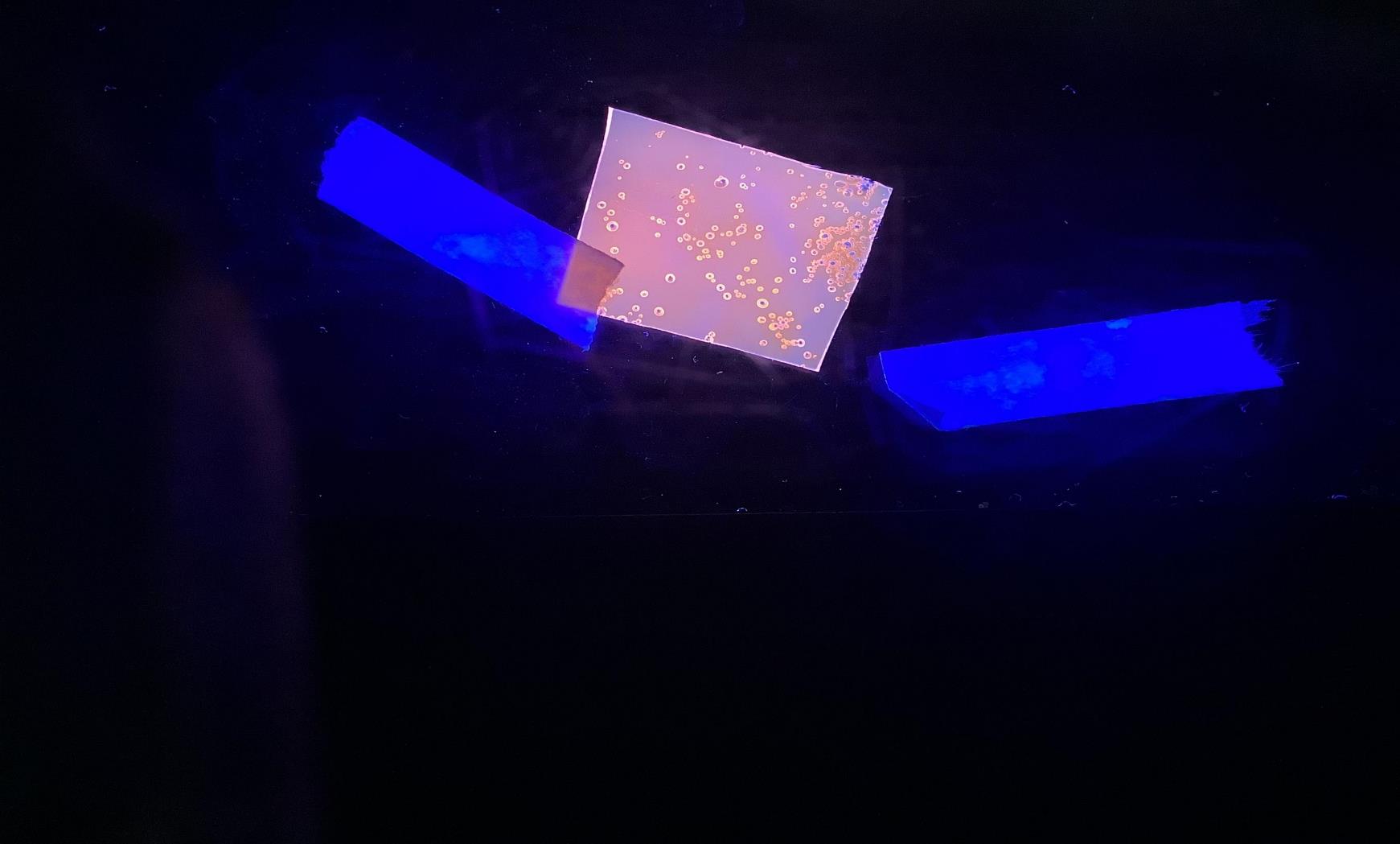 |
|  | 24.685±0.847 | 25.091±0.071 | 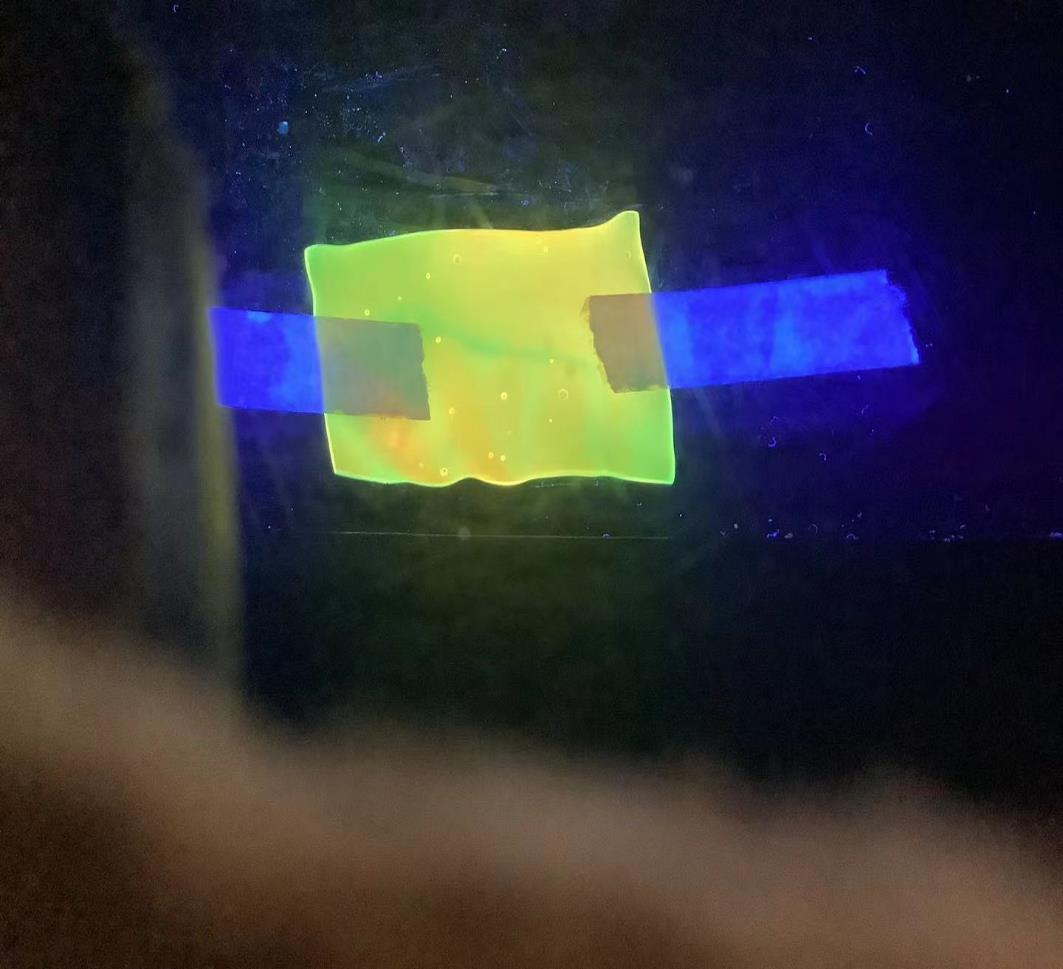 |
|  | 7.076±0.227 | - | 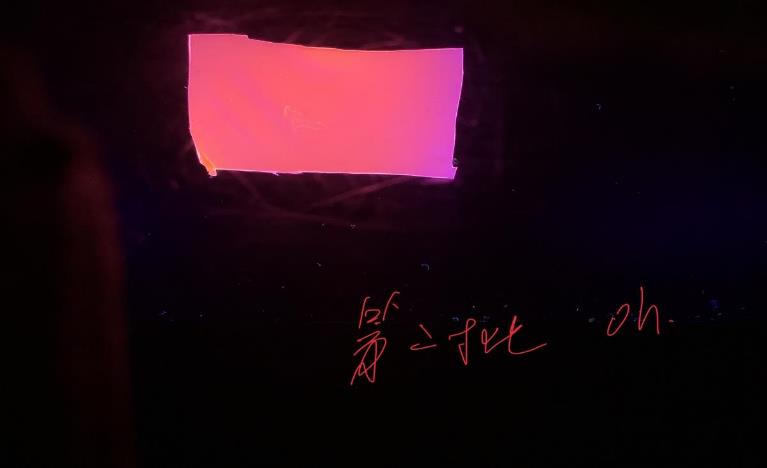 |
| 2 | 15.096±0.4 | 15.114±0.194 | 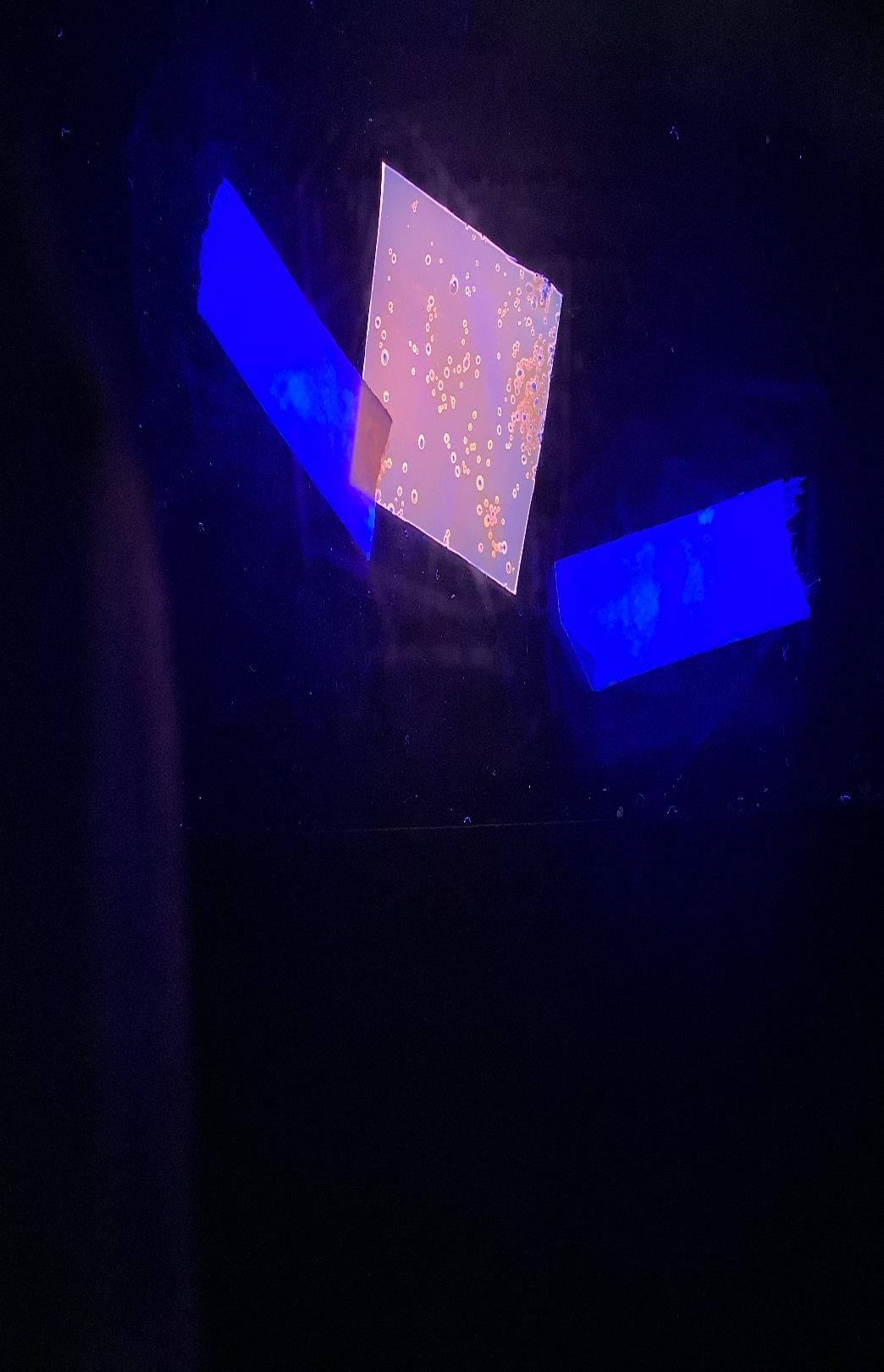 |
|  | 25.150±0.026 | 25.899±0.893 | 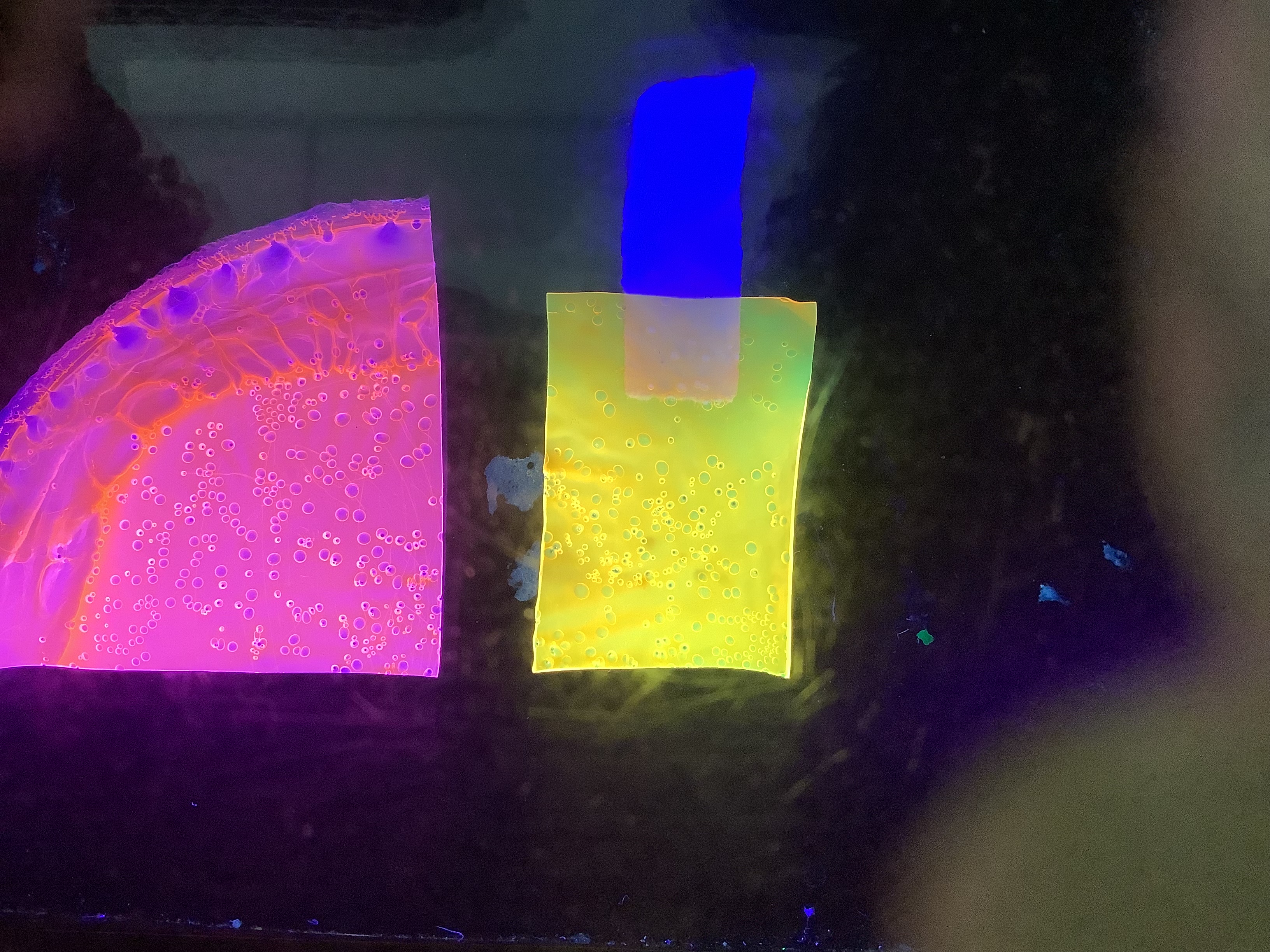 |
|  | 6.862±0.12 | - | 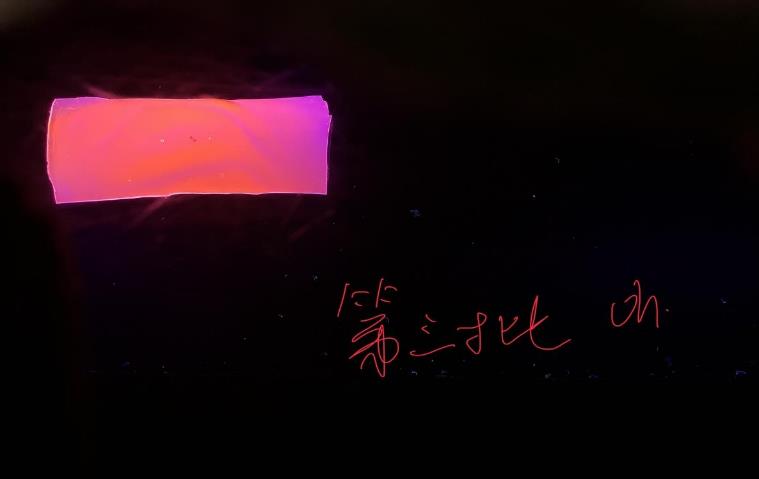 |
| 3 | 15.774±0.26 | 14.105±0.248 | 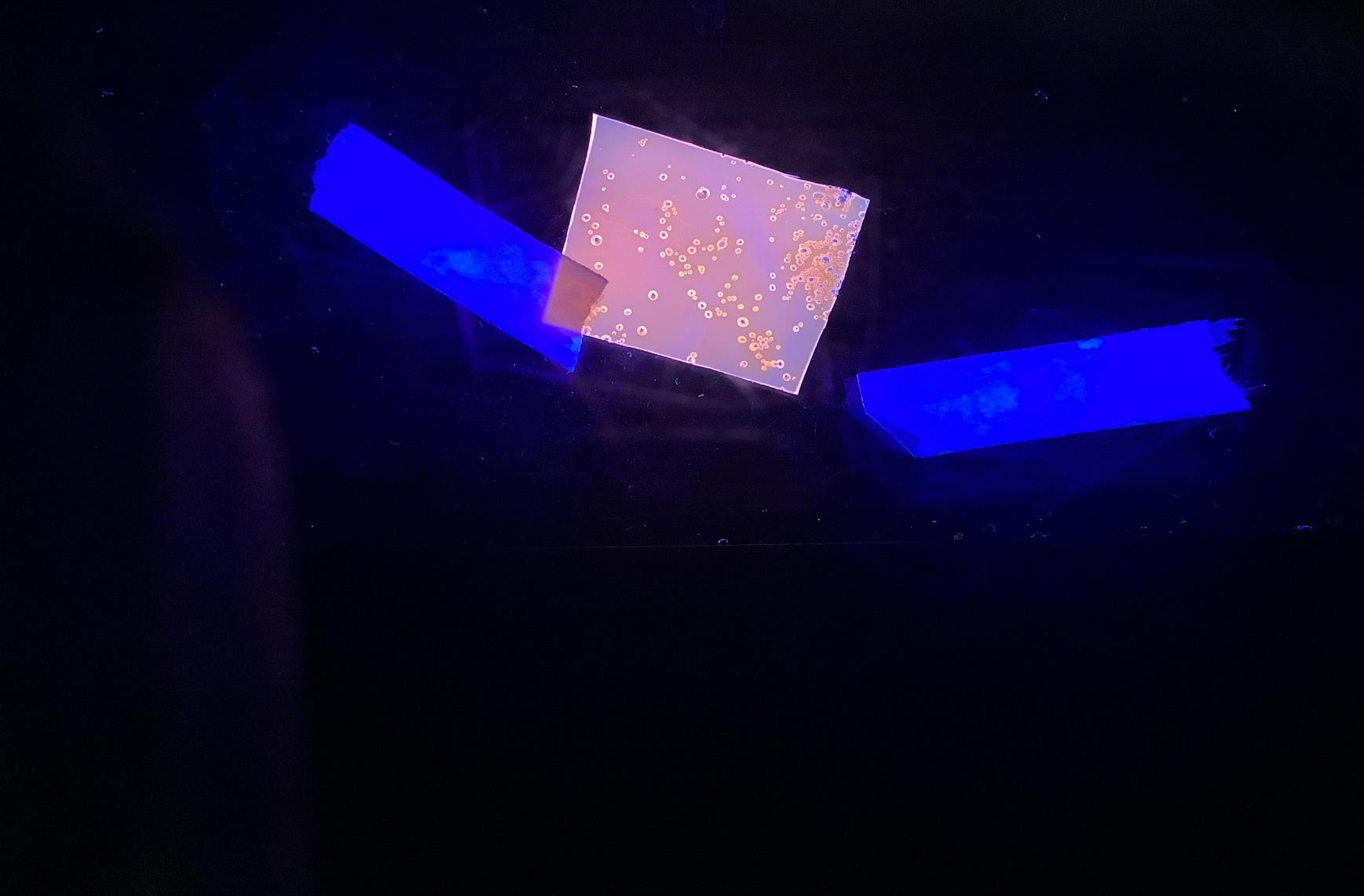 |
|  | 25.124±0.323 | 24.585±0.091 | 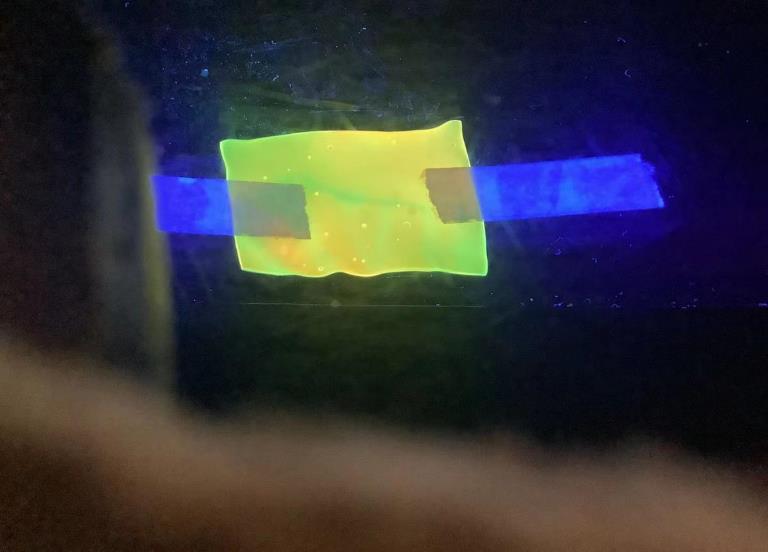 |


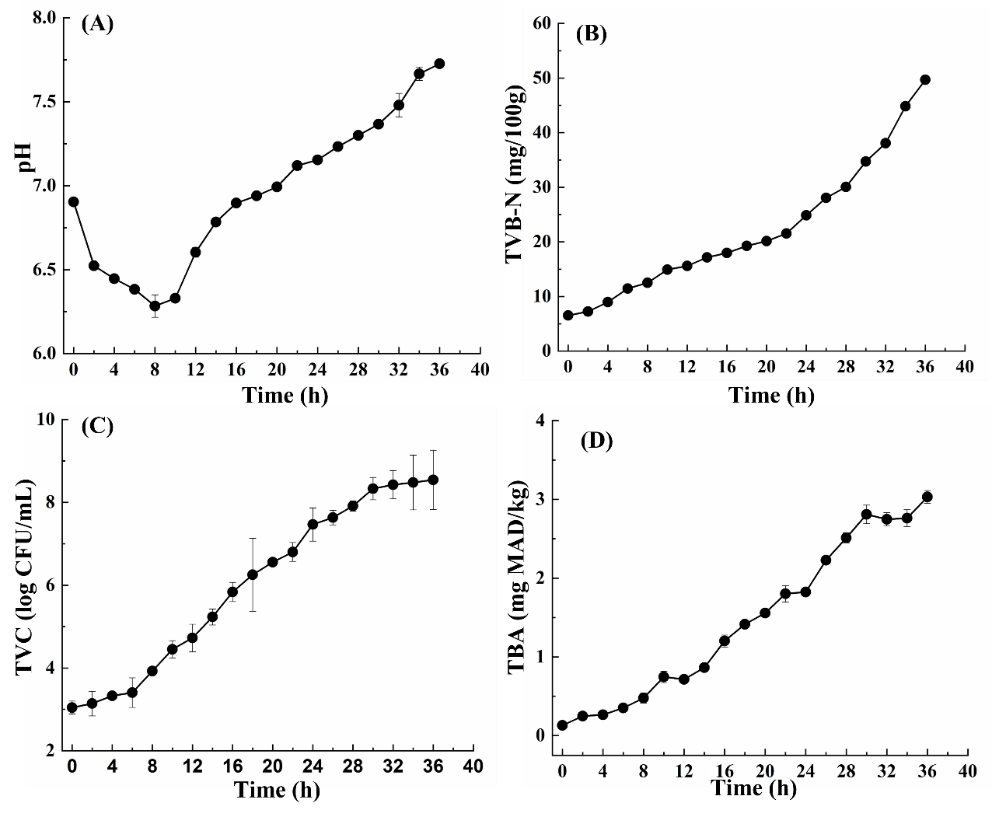


Fig. S1 Changes of (A) pH, (B) Total volatile basic nitrogen (TVB-N), (C) Total viable counts (TVC), and (D) Thiobarbital acid (TBA) values in fish samples during storage at 25 ◦C


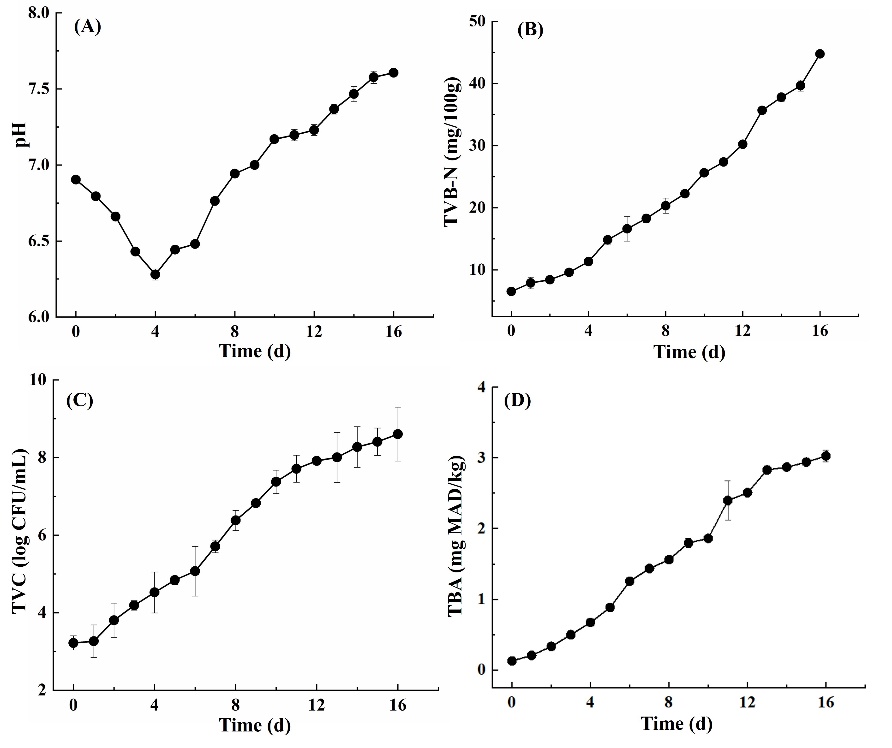


Fig. S2 Changes of (A) pH, (B) Total volatile basic nitrogen (TVB-N), (C) Total viable counts (TVC), and (D) Thiobarbituric acid (TBA) values in fish samples during storage at 4 ◦C


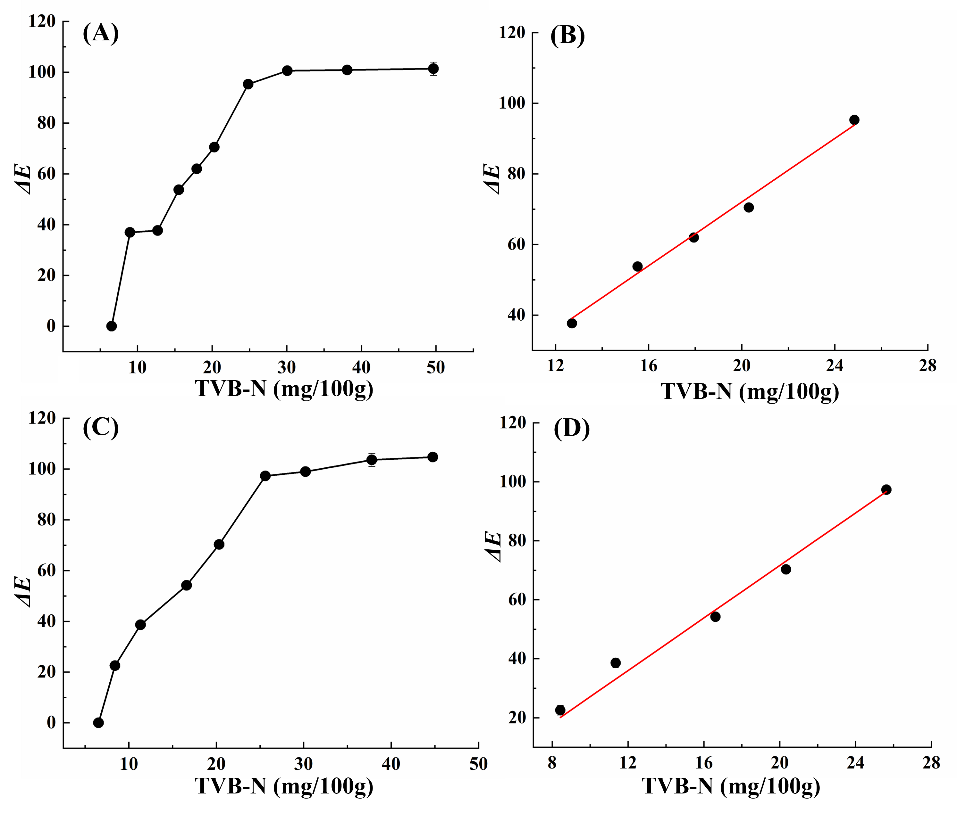


Fig. S3 Relationship and linear correlation (insert) between total volatile basic nitrogen (TVB-N) and the total color difference (*ΔE)* values at (A, B) 25 ◦C, and (C, D) 4 ◦C.
